# Homotopy perturbation method: a versatile tool to evaluate linear and nonlinear fuzzy Volterra integral equations of the second kind

**DOI:** 10.1186/s40064-016-2038-3

**Published:** 2016-03-31

**Authors:** S. Narayanamoorthy, S. P. Sathiyapriya

**Affiliations:** Department of Mathematics, Bharathiar University, Coimbatore, Tamil Nadu 641 046 India

**Keywords:** Homotopy perturbation method, Fuzzy Volterra integral equations, Numerical technique and algorithm, Approximate solutions, Error analysis

## Abstract

In this article, we focus on linear and nonlinear fuzzy Volterra integral equations of the second kind and we propose a numerical scheme using homotopy perturbation method (HPM) to obtain fuzzy approximate solutions to them. To facilitate the benefits of this proposal, an algorithmic form of the HPM is also designed to handle the same. In order to illustrate the potentiality of the approach, two test problems are offered and the obtained numerical results are compared with the existing exact solutions and are depicted in terms of plots to reveal its precision and reliability.

## Background

Integral equations find special applicability within many scientific and mathematical disciplines. Indeed modeling physical problems using integral equations with the exact parameters is not always easy but also impossible in the real problems. For this purpose, one way is using uncertainty measures for handling such lack of information. One of the most recent approaches is using fuzzy concept (Zadeh 1965). So instead of using deterministic models of integral equations, we can go in for fuzzy integral equations. Hence there occurs a need to develop mathematical models and numerical procedures that would appropriately treat general fuzzy integral equations and solve them. In this paper, we apply homotopy perturbation method (HPM) to solve both linear and nonlinear fuzzy Volterra integral equations of the second kind (FVIE-2). Many research papers dealing with fuzzy integral equations exists in open literatures and some of them are reviewed and cited here for better understanding of the present analysis. We know that solving fuzzy integral equations requires appropriate definitions of fuzzy function and fuzzy integral of a fuzzy function. We refer to the reader (Dubois and Prade 1978) for basic arithmetic operations on fuzzy numbers and he also presented the elementary fuzzy calculus (Dubois and Prade 1982) based on extension principle. Alternative approaches were later suggested by others (Goetschel and Voxman 1986; Kaleva 1987). Various methods and applications of linear and nonlinear integral equations were also reported (Wazwaz 2011). The fuzzy integral equations (Friedman et al. 1999; Subrahmanyam and Sudarsanam 1996) is one of the most important fields of fuzzy set theory. The concepts of fuzzy integral equations and fuzzy integro-differential equations have motivated a large amount of research works (Mirzaee et al. 2012; Hussain and Ali 2013a, b; Mosleh and Otadi 2013; Allahviranloo et al. 2014; Otadi and Mosleh 2014) in last decades because of its applications in scientific phenomena. Therefore investigating fuzzy integral equations by finding accurate and efficient methods for solving these equations has become an active research undertaking.

Homotopy perturbation method (He 1999) is a perturbation technique coupled with the homotopy technique was developed by He JH and was further improved by him (He 2000, 2003, 2004). In homotopy perturbation method, a complicated problem under study is continuously deformed into a simple problem which is easy to solve to obtain an approximate solution. HPM yields a very rapid convergence of the solution series in most cases, usually only few iterations lead to very accurate solutions. This method has been applied to many problems (Chun 2007; Filobello-Nino et al. 2014b) and also in particular to integral equations (Abbasbandy 2007) and fuzzy integral equations (Matinfar and Saeidy 2010). In recent past, various analytical and numerical methods were used such as Adomian decomposition method, direct computation method, series solution method, successive approximation method and conversion to integral equations. However, these methods are not easy to use and require tedious calculations. But HPM was proved (Saberi-Nadjafi and Ghorbani 2009) to be an effective and reliable tool for handling most of the linear and nonlinear differential, ordinary and partial, as well as linear and nonlinear integral equations. The main reason consists in the fact that this gives flexibility in the choice of basis functions for the solution and does not involve linear inversion operators (as compared to the Adomian decomposition method), while still retaining a simplicity that makes the method easily understandable from the standpoint of general perturbation methods.

Fuzzy integral equations are important in studying and solving a large proportion of problems in various topics in applied mathematics and it also arise in many industrial fields, such as electromagnetic fields and thermal problems. It is well-known that a large class of initial and boundary value problems can be converted to Volterra integral equations and also the problem of heat conduction with a variable heat transfer coefficient is reduced to the solution of Volterra integral equations of the second kind. Several authors applied HPM to solve linear and nonlinear partial differential equations of fractional order (Momani and Odibat 2007a, b), Volterra integral equations (Grover and Tomer 2011), singularly perturbed Volterra integral equations (Alnasr and Momani 2008) and n-th order fuzzy linear differential equations (Tapaswini and Chakraverty 2013). The reader is also referred to two recent papers where the authors (Filobello-Nino et al. 2014a; Vazquez-Leal and Sarmiento-Reyes 2015) make use of HPM in their application oriented works. In the very recent studies, it has been found that homotopy perturbation method was also used to solve fractional fisher’s equation (Hamdi Cherif et al. 2016) and a system of nonlinear chemistry problems (Ramesh Rao 2016). Taking into account all these specifications, the proposed work is mainly dealt with fuzzy Volterra integral equations. Up to authors’ knowledge no research has been carried out so far by constructing a numerical algorithm using HPM for solving fuzzy Volterra integral equations of the second kind and our work is aimed at these.

The paper is organized as follows: the fuzzy Volterra integral equations are recalled with the required theorem and the basic concept of HPM is provided. Our main results are stated in the numerical technique, which presents the detailed description of the proposed method. Besides we introduce the algorithm for solving FVIE-2. Additionally test problems are included in the section ‘Illustrative examples’ and concluding results are made over them. Finally, a brief conclusion is provided.

### *Remark*

Some of the basic notations and definitions that are not described in this paper are standard and usual. One can refer to the authors (Dubois and Prade 1978; Goetschel and Voxman 1983, 1986; Puri and Ralescu 1983, 1986; Kaleva 1987) for the notion of fuzzy numbers and its arithmetic operations, Hausdorff distance between fuzzy numbers, continuity and existence of definite integral of fuzzy functions.

## Fuzzy Volterra integral equations

The integral equations which are discussed in this section are the Volterra integral equations of the second kind. The Fredholm integral equation of the second kind (Hochstadt 1973) is given by

1 $$F\left( x \right) = f\left( x \right) + \lambda \int_{a}^{b} {k\left( {x,t} \right)F\left( t \right)dt}$$

where *λ* > 0, *a* and *b* are constants, *k*(*x*, *t*) is an arbitrary kernel function over the square *a* ≤ *x*, *t* ≤ *b* and *f*(*x*) is a function of *a* ≤ *x* ≤ *b*.

If the kernel satisfies *k*(*x*, *t*) = 0, *x* > *t*,

we obtain the Volterra integral equation of the second kind and is given by

2 $$F\left( x \right) = f\left( x \right) + \lambda \int_{a}^{x} {k\left( {x,t} \right)F\left( t \right)dt}$$

In the above equation *a* refers to constant and *x* is a variable. If *f*(*x*) is a crisp function, then the solutions of the following equations

3 $$\left( {\int_{a}^{b} {\underline{{f\left( {x;\alpha } \right)}} dx} } \right) = \int_{a}^{b} {\underline{f} \left( {x;\alpha } \right)dx}$$

4 $$\left( {\int_{a}^{b} {\overline{{f\left( {x;\alpha } \right)}} dx} } \right) = \int_{a}^{b} {\overline{f} \left( {x;\alpha } \right)dx}$$

are crisp as well, where $\left( {\underline{f} \left( {x;\alpha } \right),\overline{f} \left( {x;\alpha } \right)} \right)$ is the parametric form of *f*(*x*). However, if *f*(*x*) is a fuzzy function these equations possess fuzzy solutions.

Let *F*: *I* → *E*^1^ be a fuzzy function. The integral of *F* over *I*, denoted by $\int_{I} {F\left( x \right)dx} {\text{ or }}\int_{a}^{b} {F\left( x \right)dx}$, is defined levelwise by

$$\left[ {\int_{I} {F\left( x \right)dx} } \right]_{\alpha } = \int_{I} {F_{\alpha } \left( x \right)dx} = \left\{ {\int_{I} {f\left( x \right)dx | f:I \to R\;{\text{is a measurable function for }}F_{\alpha } } } \right\}$$

for each 0 ≤ *α* ≤ 1.

Now the parametric form of fuzzy Volterra integral equation of the second kind (FVIE-2) are as follows (Ghanbari 2010)

Let ($\underline{f} \left( {x,\alpha } \right)$, $\overline{f} \left( {x;\alpha } \right)$) and ($\underline{F} \left( {x;\alpha } \right)$, $\overline{F} \left( {x;\alpha } \right)$), be the parametric forms of *f*(*x*) and *F*(*x*) respectively, 0 ≤ *α* ≤ 1, *a* ≤ *x* ≤ *b*. Then the parametric forms of FVIE-2 are given by

5 $$\underline{F} \left( {x, \alpha } \right) = \underline{f} \left( {x,\alpha } \right) + \lambda \int_{a}^{x} {\underline{{k\left( {x, t} \right) F\left( {t, \alpha } \right)}} dt}$$

6 $$\overline{F} \left( {x, \alpha } \right) = \overline{f} \left( {x,\alpha } \right) + \lambda \int_{a}^{x} {\overline{{k\left( {x, t} \right) F\left( {t, \alpha } \right)}} dt}$$

for 0 ≤ *α* ≤ 1, where

7 $$\underline{{k\left( {x, t} \right) F\left( {t, \alpha } \right)}} = \left\{ {\begin{array}{*{20}c} {k\left( {x,t} \right) \underline{F} \left( {t, \alpha } \right)\quad k\left( {x, t} \right) \ge 0} \\ {k\left( {x, t} \right)\overline{F} \left( {t, \alpha } \right)\quad k\left( {x, t} \right) < 0} \\ \end{array} } \right.$$

and

8 $$\overline{{k\left( {x, t} \right) F\left( {t, \alpha } \right)}} = \left\{ {\begin{array}{*{20}c} {k\left( {x,t} \right) \overline{F} \left( {t, \alpha } \right)\quad k\left( {x, t} \right) \ge 0} \\ {k\left( {x, t} \right)\underline{F} \left( {t, \alpha } \right)\quad k\left( {x, t} \right) < 0} \\ \end{array} } \right.$$

The following theorem provides sufficient conditions for the existence of a unique solution to Eq. (1), where *f*(*x*) is a fuzzy function.

### **Theorem**

(Wu and Ma 1990) *Let*$k\left( {x, t} \right)$*be continuous for a* *≤* *x, t* *≤* *b, λ* *>* 0*, and f*(*x*) *is a fuzzy continuous function of x, a* *≤* *x* *≤* *b. If*$\lambda < \frac{1}{{M\left( {b - a} \right)}}$*, where*$$M = \begin{array}{*{20}c} {\hbox{max} } \\ {\left( {a \le x, t \le b} \right)} \\ \end{array} \left| {k\left( {x,t} \right)} \right|$$, *then the iterative procedure*

9 $$\begin{aligned} F_{0} \left( x \right) & = f\left( x \right), \\ F_{k} \left( x \right) & = f\left( x \right) + \lambda \int_{a}^{b} {k\left( {x, t} \right)F_{k - 1} \left( t \right)dt} , \quad k \ge 1, \\ \end{aligned}$$

*converges to the unique solution of Eq.* (1)*. Specifically,*

10 $$\begin{array}{*{20}c} {\sup } \\ {\left( {a \le x \le b} \right)} \\ \end{array} D(F\left( x \right),\; F_{k} \left( x \right)) \le \frac{{L^{k} }}{1 - L} \begin{array}{*{20}c} {\sup } \\ {\left( {a \le x \le b} \right)} \\ \end{array} D\left( {F_{0} \left( x \right),\;F_{1} \left( x \right)} \right),$$

*where L* *=* *λM*(*b* *−* *a*)*. This refers that F*_*k*_(*x*) *converges uniformly in x to F*(*x*)*, i.e., given arbitrary ɛ* *>* *0 we can find N such that*

$$D\left( {F\left( x \right),F_{k} \left( x \right)} \right) < \varepsilon ,\quad a \le x \le b,\; k > N.$$

*The proof this theorem can be easily extended for fuzzy Volterra integral equations of the second kind, i.e., for Eq.* (2) *where f*(*x*) *is a fuzzy function as well.*

## Basic concept of homotopy perturbation method and its application to Volterra integral equations

For convenience of the reader, we present the review of the HPM. The essential idea of this method is to introduce a homotopy parameter, say *p*, which takes the values from 0 to 1. When *p* = 0, the system of equation usually reduces to a sufficiently simplified form, which normally admits a rather simple solution. As *p* gradually increases to 1, the system goes through a sequence of deformation, the solution of each of which is close to that at the previous stage of deformation. Eventually at *p* = 1, the system takes the original form of the equation and final stage of deformation gives the desired solution.

To figure out how HPM works, consider the nonlinear integral equation

11 $$L\left( u \right) + N\left( u \right) = f\left( r \right),\quad r\epsilon{\varvec\varOmega },$$

with boundary conditions $B\left( {u, \frac{\partial u}{\partial n}} \right) = 0$, $r \epsilon \varGamma$,

where *L* is a linear operator, *N* is a nonlinear operator, *B* is a boundary operator, *Γ* is the boundary of the domain $\varvec{\varOmega}$ and *f*(*r*) is a known analytic function. In order to use the HPM, a suitable construction of homotopy is of vital importance. If *L*(*u*) = 0 with some possible unknown parameter can best describe the original nonlinear system. Generally, a homotopy can be constructed as (He 1999, 2000)

12 $$H\left( {U,p} \right) = \left( {1 - p} \right)\left[ {L\left( U \right) - L\left( {u_{0} } \right)} \right] + p\left[ {L\left( U \right) + N\left( U \right) - f\left( r \right)} \right] = 0$$

or

13 $$H\left( {U,p} \right) = L\left( U \right) - L\left( {u_{0} } \right) + p\left[ {L\left( {u_{0} } \right) + N\left( U \right) - f\left( r \right)} \right] = 0 ,$$

where $$p \epsilon [0, 1]$$ and $$r \epsilon {\varvec\varOmega }$$.

The nonlinear Volterra integral equation is given by

14 $$\gamma \left( x \right) = f\left( x \right) + \int_{a}^{x} {k\left( {x, t} \right)\left\{ {R\left( {\gamma \left( t \right)} \right) + N\left( {\gamma \left( t \right)} \right)} \right\}dt} ,\quad a \le x,\;t \le b$$

where *γ*(*x*) is an unknown function that will be determined, *k*(*x*, *t*) is the kernel of the integral equation, *f*(*x*) is an analytic function, *R*(*γ*) and *N*(*γ*) are linear and nonlinear function of *γ* respectively.

To illustrate the HPM, we reconstitute Eq. (14) as

15 $$L\left( u \right) = u\left( x \right) - f\left( x \right) - \int_{a}^{x} {k\left( {x,t} \right)\left\{ {R\left( {u\left( t \right)} \right) + N\left( {u\left( t \right)} \right)} \right\}dt} ,$$

with the solution *u*(*x*) = *γ*(*x*) and we define the homotopy *H*(*U*, *p*) by

16 $$H\left( {U, 0} \right) = F\left( u \right),\quad H\left( {U, 1} \right) = L\left( u \right),$$

where *F*(*u*) is a functional operator with solution, say *u*_0_, which can be obtained easily. We can choose a homotopy

17 $$H\left( {u,p} \right) = \left( {1 - p} \right)F\left( u \right) + pL\left( u \right) = 0,$$

and continuously trace an implicitly defined curve from a starting point $H\left( {u_{0} , 0} \right)$ to a solution function *H*(*γ*, 1). The embedding parameter *p* monotonically increases from 0 to 1 as the trivial problem *F*(*u*) = 0 continuously deformed to the original problem *L*(*u*) = 0. The embedded parameter $p \epsilon [0, 1]$ can be considered as an expanding parameter.

18 $$u = \mathop \sum \limits_{n = 0}^{\infty } p^{n} u_{n}$$

When *p* → 1, Eq. (17) corresponds to Eq. (15) and Eq. (18) becomes the approximate solution of Eq. (14), i.e.,

19 $$\gamma \left( x \right) = \begin{array}{*{20}c} {\lim } \\ {p \to 1} \\ \end{array} u = \mathop \sum \limits_{n = 0}^{\infty } u_{n}$$

The above series is convergent in most of the cases and also the rate of convergence depends on *L*(*u*) (He 2000).

In other words, it is very natural to assume that the solution of [Eqs. (16) and (17)] can be expressed as

20 $$\gamma = u_{0} + p^{1} u_{1} + p^{2} u_{2} + \cdots$$

Equating the terms with identical powers of *p*, we get

21 $$\begin{aligned} & p^{0} : u_{0} \left( x \right) = f\left( x \right) \\ & p^{1} : u_{1} \left( x \right) = \int_{a}^{x} {k\left( {x,t} \right)u_{0} \left( t \right)dt} \\ & \vdots \\ & p^{n} : u_{n} \left( x \right) = \int_{a}^{x} {k\left( {x,t} \right)u_{n - 1} \left( t \right)dt} \; \; {\text{and so on}}. \\ \end{aligned}$$

Therefore when *p* = 1, the approximate solution of above equation can be readily obtained as follows

22 $$\gamma = \begin{array}{*{20}c} {\lim } \\ {p \to 1} \\ \end{array} u_{0} + u_{1} + u_{2} + \cdots$$

The above series is convergent for most cases.

## Proposed numerical technique for solving FVIE-2 using HPM

As HPM is considered as a combination of the classical perturbation technique and the homotopy (whose origin is in the topology), but not restricted to small parameters as occur with traditional perturbation methods, because HPM neither requires small parameter nor linearization, but only few iterations to obtain highly accurate solution. Hence we introduce the recursive scheme for solving FVIE-2 as follows

In view of homotopy, we can define the following convex homotopy,

23 $$\left\{ {\begin{array}{*{20}c} {\left( {1 - p} \right)\left[ { \underline{V} \left( {x, \alpha } \right) - \underline{u}_{0} \left( {x,\alpha } \right)} \right] + p\left[ {\underline{V} \left( {x, \alpha } \right) - \underline{f} \left( {x, \alpha } \right) - \int_{a}^{x} {k\left( {x,t} \right)\underline{V} \left( {x, \alpha } \right)dt} } \right] = 0} \\ { \left( {1 - p} \right)\left[ { \bar{V}\left( {x, \alpha } \right) - \bar{u}_{0} \left( {x,\alpha } \right)} \right] + p\left[ { \bar{V}\left( {x, \alpha } \right) - \bar{f}\left( {x, \alpha } \right) - \int_{a}^{x} {k\left( {x,t} \right)\bar{V}\left( {x, \alpha } \right)dt} } \right] = 0} \\ \end{array} } \right.$$

where *α* is the fuzzy parameter (0 ≤ *α* ≤ 1).

The solution of Eq. (23) is assumed in the following form

24 $$\left\{ {\begin{array}{*{20}c} {\underline{V} \left( {x, \alpha } \right) = \mathop \sum \limits_{i = 0}^{\infty } p^{i} \underline{V}_{i} \left( {x, \alpha } \right)} \\ {\bar{V}\left( {x, \alpha } \right) = \mathop \sum \limits_{i = 0}^{\infty } p^{i} \bar{V}_{i} \left( {x, \alpha } \right)} \\ \end{array} } \right.$$

where $\left( {\underline{V}_{i} , \bar{V}_{i} } \right) \forall i$ are unknown functions to be determined.

The initial approximation is taken as

25 $$\left\{ {\begin{array}{*{20}c} {\underline{V} \left( {0, \alpha } \right) = \underline{u}_{0} \left( {0,\alpha } \right) = \underline{f} \left( {x, \alpha } \right)} \\ {\bar{V}\left( {0, \alpha } \right) = \bar{u}_{0} \left( {0,\alpha } \right) = \bar{f}\left( {x, \alpha } \right)} \\ \end{array} } \right.$$

Substituting Eqs. (24) and (25) in Eq. (23) reduces to

26 $$\left\{ {\begin{array}{*{20}c} {\underline{V} \left( {x, \alpha } \right) = \underline{u}_{0} \left( {x,\alpha } \right) + p\int_{a}^{x} {k\left( {x,t} \right)\underline{V} \left( {x, \alpha } \right)dt} } \\ {\bar{V}\left( {x, \alpha } \right) = \bar{u}_{0} \left( {x,\alpha } \right) + p\int_{a}^{x} {k\left( {x,t} \right)\bar{V}\left( {x, \alpha } \right)dt} } \\ \end{array} } \right.$$

Equating the coefficients with like powers of *p*, we yield the following iterations

27 $$p^{0} :\left\{ {\begin{array}{*{20}c} {\underline{V} \left( {0, \alpha } \right) = \underline{f} \left( {x, \alpha } \right)} \\ {\bar{V}\left( {0, \alpha } \right) = \bar{f}\left( {x, \alpha } \right)} \\ \end{array} } \right.$$

28 $$p^{1} :\left\{ {\begin{array}{*{20}c} {\underline{V}_{1} \left( {x, \alpha } \right) = \int_{a}^{x} {k\left( {x,t} \right)\underline{f} \left( {t, \alpha } \right)dt} } \\ { \bar{V}_{1} \left( {x, \alpha } \right) = \int_{a}^{x} {k\left( {x,t} \right)\bar{f}\left( {t, \alpha } \right)dt} } \\ \end{array} } \right.$$

29 $$\begin{aligned} & p^{2} :\left\{ {\begin{array}{*{20}c} {\underline{V}_{2} \left( {x, \alpha } \right) = \mathop \smallint \nolimits_{a}^{x} k\left( {x,t} \right)\underline{V}_{1} \left( {t, \alpha } \right)dt} \\ { \bar{V}_{2} \left( {x, \alpha } \right) = \mathop \smallint \nolimits_{a}^{x} k\left( {x,t} \right) \bar{V}_{1} \left( {t, \alpha } \right)dt} \\ \end{array} } \right. \hfill \\ & \vdots \hfill \\ & {\text{and so on}} .\hfill \\ \end{aligned}$$

Finally, the solution of FVIE-2 is given by

30 $$\left\{ {\begin{array}{*{20}c} { \underline{u} \left( {x, \alpha } \right) = \begin{array}{*{20}c} {lim} \atop {p \to 1} \\ \end{array} \underline{V} \left( {x, \alpha } \right) = \mathop \sum \limits_{i = 0}^{\infty } \underline{V}_{i} \left( {x, \alpha } \right)} \\ {\bar{u}\left( {x, \alpha } \right) = \begin{array}{*{20}c} {lim} \atop {p \to 1} \\ \end{array} \bar{V}\left( {x, \alpha } \right) = \mathop \sum \limits_{i = 0}^{\infty } \bar{V}_{i} \left( {x, \alpha } \right)} \\ \end{array} } \right.$$

In general, we obtain the following iteration formulae

31 $$p^{i + 1} :\left\{ \begin{aligned} \underline{V}_{i + 1} \left( {x, \alpha } \right) = \mathop \smallint \nolimits_{a}^{x} k\left( {x,t} \right)\underline{V}_{i} \left( {t, \alpha } \right)dt \hfill \\ \bar{V}_{i + 1} \left( {x, \alpha } \right) = \mathop \smallint \nolimits_{a}^{x} k\left( {x,t} \right) \bar{V}_{i} \left( {t, \alpha } \right)dt \hfill \\ \end{aligned} \right.$$

These relations [Eq. (31)] will enable us to determine the components $\underline{u} \left( {x, \alpha } \right)$ and $\bar{u}\left( {x, \alpha } \right)$ recursively for *i* ≥ 0.

## Algorithm of the approach

On the basis of our proposed results using HPM, which are discussed in the above section, we convert to a numerical HPM algorithm that is one of the main results of this paper. Hence, in an algorithmic form, HPM can be expressed and implemented as follows.

The Eqs. (5) and (6) with the iteration index *i* ≥ 0, and a appropriate value for the tolerance (here $Tol = 10^{ - 5}$).

## Main steps

Step 1: For reckoning the initial data, let $\underline{V}_{0} = \underline{V}$ and $\bar{V}_{0}$ = $\bar{V}$ where *x* = 0
Step 2: Use the recursive relations [Eq. (31)] to compute the values of $\underline{V}_{i + 1} \left( x \right)$ by using $\underline{V}_{i} \left( x \right)$ and $\bar{V}_{i + 1} \left( x \right)$ by using $\bar{V}_{i} \left( x \right)$
Step 3: If $\left\{ {\hbox{max} \left| {\underline{V}_{i + 1} \left( x \right) - \underline{V}_{i} \left( x \right)} \right| < Tol, max\left| { \bar{V}_{i + 1} \left( x \right) - \bar{V}_{i} \left( x \right)} \right| < Tol} \right\}$, then go to step 4, else set *i* = *i* + 1 and go to step 2
Step 4: Print $\underline{u} \left( x \right) = \sum\nolimits_{i = 0}^{\infty } {\underline{V}_{i} }$ and $\bar{u}\left( x \right) = \sum\nolimits_{i = 0}^{\infty } {\bar{V}_{i} }$ as the approximates of the exact solutions

## Illustrative examples

To give a clear overview and to demonstrate the efficiency of the homotopy perturbation method, we implement the proposed numerical technique by solving a linear FVIE-2 and a nonlinear FVIE-2 with known exact solutions.

## Linear fuzzy Volterra integral equations of the second kind

### *Example 1*

We consider the linear FVIE-2 given by

$$F\left( x \right) = f\left( x \right) + \lambda \int_{a}^{x} {k\left( {x, t} \right)F\left( t \right)dt} ,\quad 0 \le x \le 1, \, 0 \le t \le x$$

where *λ* = 1, *a* = 0, *k*(*x*, *t*) = *sinhx* and

$$\begin{aligned} f\left( x \right) & = \left( {\underline{f} \left( {x, \alpha } \right), \bar{f}\left( {x, \alpha } \right)} \right) \\ & = \left\{ {\left( {\cosh x + 1 - \cosh^{2} x} \right)\left( {\alpha^{2} + \alpha } \right), \left( {\cosh x + 1 - \cosh^{2} x} \right)\left( {4 - \alpha^{3} - \alpha } \right)} \right\},\quad 0 \le \alpha \le 1 \\ \end{aligned}$$

The exact solution in this case is given by

$$\underline{F} \left( {x, \alpha } \right) = \underline{u} \left( {x,\alpha } \right) = coshx\left( {\alpha^{2} + \alpha } \right)$$

$$\bar{F}\left( {x, \alpha } \right) = \bar{u}\left( {x, \alpha } \right) = coshx\left( {4 - \alpha^{3} - \alpha } \right)$$

Here we have

$$\underline{{k\left( {x, t} \right) F\left( {t, \alpha } \right)}} = \left( {sinhx} \right)\underline{F} \left( {t,\alpha } \right)$$

$$\overline{{k\left( {x, t} \right) F\left( {t, \alpha } \right)}} = \left( {sinhx} \right)\bar{F}\left( {t, \alpha } \right)$$

To solve the given equation by HPM we construct a convex homotopy as follows

$$H\left( {\underline{V} ,p,\alpha } \right) = \underline{V} \left( {x, \alpha } \right) - \left( {coshx + 1 - cosh^{2} x} \right)\left( {\alpha^{2} + \alpha } \right) + p\int_{a}^{x} {k\left( {x,t} \right)\underline{V} \left( {t, \alpha } \right)dt}$$

$$H\left( {\bar{V},p,\alpha } \right) = \bar{V}\left( {x, \alpha } \right) - \left( {coshx + 1 - cosh^{2} x} \right)\left( {4 - \alpha^{3} - \alpha } \right) + p\int_{a}^{x} {k\left( {x,t} \right)\bar{V}\left( {t, \alpha } \right)dt}$$

Now we make use of the iteration formulae [Eq. (31)] and applying the numerical algorithm, the HPM solution series with few iterative terms are as follows

$p^{0} :( \underline{V}_{0} \left( {x,\alpha } \right), \bar{V}_{0} \left( {x,\alpha } \right)$) is the initial fuzzy approximations,where

$$\underline{V}_{0} \left( {x,\alpha } \right) = \underline{f} \left( {x, \alpha } \right) = \left( {coshx + 1 - cosh^{2} x} \right)\left( {\alpha^{2} + \alpha } \right)$$

$$\bar{V}_{0} \left( {x,\alpha } \right) = \bar{f}\left( {x, \alpha } \right) = \left( {coshx + 1 - cosh^{2} x} \right)\left( {4 - \alpha^{3} - \alpha } \right)$$

$$p^{1} :( \underline{V}_{1} \left( {x,\alpha } \right), \bar{V}_{1} \left( {x,\alpha } \right)),$$

where

$$\underline{V}_{1} \left( {x,\alpha } \right) = \frac{ - 1}{4}\alpha \left( {1 + \alpha } \right)sinhx\left( { - 2x - 4\,sinhx + sinh2x} \right)$$

$$\bar{V}_{1} \left( {x,\alpha } \right) = \frac{1}{4}\left( { - 4 + \alpha + \alpha^{3} } \right)sinhx\left( { - 2x - 4\,sinhx + sinh2x} \right)$$

In the same way, the iterations $p^{2} :\left( { \underline{V}_{2} \left( {x,\alpha } \right), \bar{V}_{2} \left( {x,\alpha } \right)} \right)$ and $p^{3} :\left( {\underline{V}_{3} \left( {x,\alpha } \right), \bar{V}_{3} \left( {x,\alpha } \right)} \right)$ were also computed for calculating fuzzy approximate solutions which could not be stated here as the iterations contain very long expressions.

We approximate $\underline{F} \left( {x,\alpha } \right)$ and $\bar{F}\left( {x,\alpha } \right)$ by setting *p* = 1 and obtain the following fuzzy solutions, which are given by

$$\begin{aligned} \underline{F} \left( {x,\alpha } \right) & = \underline{u} \left( {x,\alpha } \right) = \mathop \sum \limits_{i = 0}^{3} \underline{V}_{i} (x,\alpha ) \\ & = \left( {coshx + 1 - cosh^{2} x} \right)\left( {\alpha^{2} + \alpha } \right) \\ & \quad + \left( {\frac{ - 1}{4}\alpha \left( {1 + \alpha } \right)sinhx( - 2x - 4\,sinhx + sinh2x)} \right) + \cdots \\ \end{aligned}$$

and

$$\begin{aligned} \bar{F}\left( {x,\alpha } \right) & = \bar{u}\left( {x, \alpha } \right) = \mathop \sum \limits_{i = 0}^{3} \bar{V}_{i} \left( {x,\alpha } \right) \\ & = \left( {coshx + 1 - cosh^{2} x} \right)\left( {4 - \alpha^{3} - \alpha } \right) \\ & \quad + \left( {\frac{1}{4}\left( { - 4 + \alpha + \alpha^{3} } \right)sinhx( - 2x - 4\,sinhx + sinh2x)} \right) + \cdots \\ \end{aligned}$$

### Error analysis

Absolute errors are computed as

$$\underline{E} \left( {x,\alpha } \right) = \left| {coshx\left( {\alpha^{2} + \alpha } \right) - \underline{u} \left( {x,\alpha } \right)} \right|$$

$$\bar{E}\left( {x,\alpha } \right) = \left| {coshx\left( {4 - \alpha^{3} - \alpha } \right) - \bar{u}\left( {x,\alpha } \right)} \right|$$

**Fig. 1:**
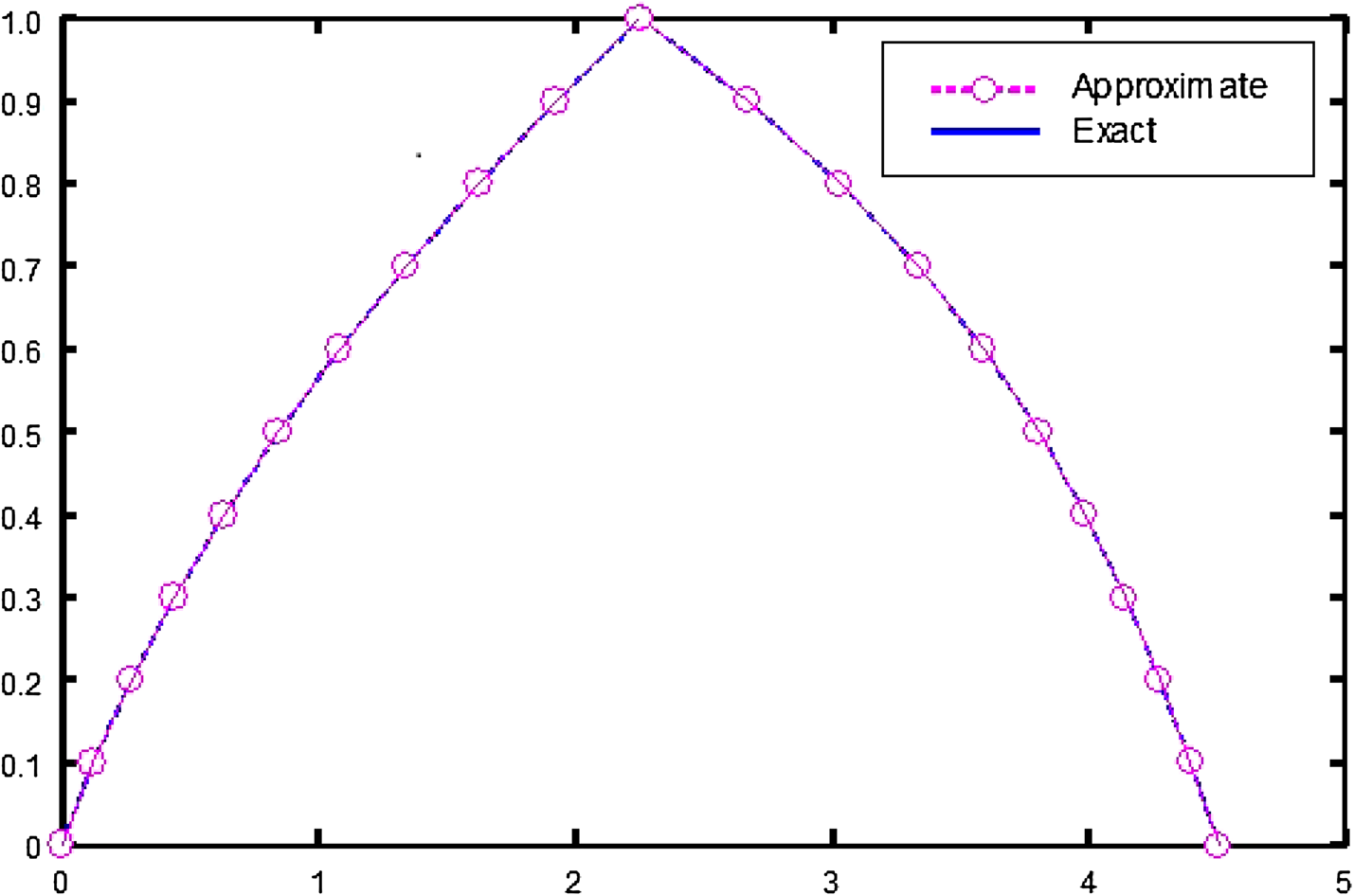
Plots of exact and approximate solutions of Example 1

The components of iteration formulae [Eq. (31)] were obtained by the Mathematica program (Mathematica package version 5.2) according to the numerical algorithm where the tolerance is given a suitable positive value. The numerical results of the obtained approximate solutions are compared with the exact solutions for different *α***-**values and are presented in Table 1. Moreover exact and approximate solutions are shown graphically in Fig. 1.

**Table 1:** Exact and approximate solutions with four iterations at *x* = 0.5

*α*	Exact solution		Approximate solution		Error	
	$\underline{u} \left( {x,\alpha } \right)$	$\bar{u}\left( {x,\alpha } \right)$	$\underline{u} \left( {x,\alpha } \right)$	$\bar{u}\left( {x,\alpha } \right)$	$\underline{E} \left( {x,\alpha } \right)$	$\bar{E}\left( {x,\alpha } \right)$
0	0	4.51050	0	4.51034	0	0.00016
0.1	0.124039	4.39661	0.124034	4.39645	0.000005	0.00016
0.2	0.270630	4.27596	0.270620	4.27580	0.000010	0.00016
0.3	0.439774	4.14177	0.439758	4.14162	0.000016	0.00015
0.4	0.631471	3.98729	0.631447	3.98714	0.000024	0.00015
0.5	0.845719	3.80574	0.845688	3.80560	0.000031	0.00014
0.6	1.082520	3.59036	1.082480	3.59023	0.000040	0.00013
0.7	1.341870	3.33439	1.341820	3.33427	0.000050	0.00012
0.8	1.623780	3.03106	1.623720	3.03095	0.000060	0.00011
0.9	1.928240	2.67360	1.928170	2.67350	0.000070	0.00010
1	2.255250	2.25525	2.255170	2.25517	0.000080	0.00008

## Nonlinear fuzzy Volterra integral equations of the second kind

### *Example 2*

We consider the nonlinear FVIE-2 given by

$$F\left( x \right) = f\left( x \right) + \lambda \int_{a}^{a} {k\left( {x, t} \right)F^{2} \left( t \right)dt} ,\quad 0 \le x \le 1, \; 0 \le t \le x$$

where *λ* = 1, *a* = 0, *k*(*x*, *t*) = *x*^2^(1 − 2*t*) and

$$\begin{aligned} f\left( x \right) & = \left( {\underline{f} \left( {x, \alpha } \right), \bar{f}\left( {x, \alpha } \right)} \right) \\ & = \left\{ {\left( {\left( {2 - \alpha } \right)^{2} \left( { \frac{1}{2}x^{6} + x^{5} - x^{3} + \frac{11}{32}x^{2} } \right) - \frac{11}{32}x^{2} + \alpha x + \alpha } \right)} \right., \\ & \quad \left. {\left( {\alpha^{2} \left( {\frac{1}{2}x^{6} + x^{5} - x^{3} + \frac{11}{32}x^{2} } \right) + \left( {2 - \alpha } \right)\left( {\frac{ - 11}{32}\left( {2 - \alpha } \right)x^{2} + x + 1} \right)} \right)} \right\},\quad 0 \le \alpha \le 1 \\ \end{aligned}$$

The exact solution in this case is given by

$$\underline{F} \left( {x, \alpha } \right) = \underline{u} \left( {x,\alpha } \right) = \alpha \left( {x + 1} \right)$$

$$\bar{F}\left( {x, \alpha } \right) = \bar{u}\left( {x, \alpha } \right) = \left( {2 - \alpha } \right)\left( {x + 1} \right)$$

Here we have

$$\underline{{k\left( {x, t} \right) F^{2} \left( {t, \alpha } \right)}} = x^{2} \left( {1 - 2t} \right)\underline{F}^{2} \left( {t,\alpha } \right)$$

$$\overline{{k\left( {x, t} \right) F^{2} \left( {t, \alpha } \right)}} = x^{2} \left( {1 - 2t} \right)\bar{F}^{2} \left( {t, \alpha } \right)$$

To solve the given equation by HPM we construct a convex homotopy as follows

$$\begin{aligned} H\left( {\underline{V} ,p,\alpha } \right) & = \underline{V} \left( {x, \alpha } \right) - \left( {\left( {2 - \alpha } \right)^{2} \left( {\frac{1}{2}x^{6} + x^{5} - x^{3} + \frac{11}{32}x^{2} } \right) - \frac{11}{32}x^{2} + \alpha x + \alpha } \right) \\ & \quad + p\mathop \smallint \nolimits_{a}^{x} k\left( {x,t} \right) \underline{V}^{2} \left( {t, \alpha } \right)dt \\ \end{aligned}$$

$$\begin{aligned} H\left( {\bar{V},p,\alpha } \right) & = \bar{V}\left( {x, \alpha } \right) - \left( {\alpha^{2} \left( {\frac{1}{2}x^{6} + x^{5} - x^{3} + \frac{11}{32}x^{2} } \right) + \left( {2 - \alpha } \right)\left( {\frac{ - 11}{32}\left( {2 - \alpha } \right)x^{2} + x + 1} \right)} \right) \\ & \quad + p\mathop \smallint \nolimits_{a}^{x} k\left( {x,t} \right)\bar{V}^{2} \left( {t, \alpha } \right)dt \\ \end{aligned}$$

Now we make use of the iteration formulae [Eq. (31)] and applying the numerical algorithm, the HPM solution series with few iterative terms are as follows

$p^{0} :\left( {\underline{V}_{0} \left( {x,\alpha } \right), \bar{V}_{0} \left( {x,\alpha } \right)} \right)$ is the initial fuzzy approximations,

where

$$\underline{V}_{0} \left( {x,\alpha } \right) = \underline{f} \left( {x, \alpha } \right) = \left( {2 - \alpha } \right)^{2} \left( { \frac{1}{2}x^{6} + x^{5} - x^{3} + \frac{11}{32}x^{2} } \right) - \frac{11}{32}x^{2} + \alpha x + \alpha$$

$$\bar{V}_{0} \left( {x,\alpha } \right) = \bar{f}\left( {x, \alpha } \right) = \alpha^{2} \left( {\frac{1}{2}x^{6} + x^{5} - x^{3} + \frac{11}{32}x^{2} } \right) + \left( {2 - \alpha } \right)\left( {\frac{ - 11}{32}\left( {2 - \alpha } \right)x^{2} + x + 1} \right)$$

$$p^{1} :(\underline{V}_{1} \left( {x,\alpha } \right), \bar{V}_{1} \left( {x,\alpha } \right), )$$

where

$$\begin{aligned} & \underline{V}_{1} \left( {x,\alpha } \right) \\ & \quad = x^{2} \left( {\alpha^{2} x + \frac{1}{48}\alpha \left( {33 + \alpha \left( { - 92 + 11\alpha } \right)} \right)x^{3} } \right. \\ & \quad \quad - \frac{1}{64}\alpha \left( {161 + \alpha \left( { - 140 + 43\alpha } \right)} \right)x^{4} \\ & \quad \quad + \frac{1}{5120}\left( {1089 + \alpha \left( {1064 + \alpha \left( {102 + \alpha \left( { - 328 + 121\alpha } \right)} \right)} \right)} \right)x^{5} \\ & \quad \quad - \frac{1}{3072}\left( {5313 + \alpha \left( { - 25048 + \alpha \left( {23046 + \alpha \left( { - 6856 + 473\alpha } \right)} \right)} \right)} \right)x^{6} \\ & \quad \quad + \frac{1}{56}\left( { - 2 + \alpha } \right)^{2} \left( { - 1 + \alpha } \right)\left( { - 65 + 19\alpha } \right)x^{7} \\ & \quad \quad - \frac{1}{128}\left( { - 2 + \alpha } \right)^{2} \left( {95 + \alpha \left( { - 4 + 21\alpha } \right)} \right)x^{8} \\ & \quad \quad - \frac{1}{288}\left( { - 2 + \alpha } \right)^{2} \left( {355 + \alpha \left( { - 324 + 97\alpha } \right)} \right)x^{9} \\ & \quad \quad + \frac{1}{160}\left( { - 2 + \alpha } \right)^{2} \left( {159 + 37\left( { - 4 + \alpha } \right)\alpha } \right)x^{10} + \frac{3}{11}\left( { - 2 + \alpha } \right)^{4} x^{11} \\ & & \quad \quad - \left. {\frac{1}{12}\left( { - 2 + \alpha } \right)^{4} x^{12} - \frac{7}{52}\left( { - 2 + \alpha } \right)^{4} x^{13} - \frac{1}{28}\left( { - 2 + \alpha } \right)^{4} x^{14} } \right) \\ \end{aligned}$$

$$\begin{aligned} & \bar{V}_{1} \left( {x,\alpha } \right) \\ & \quad = \frac{ - 1}{64}x^{2} \left( { - 64(2 + \alpha )^{2} x + \frac{16}{3}\left( {2 + \alpha } \right)\left( { - 35 + 23\alpha } \right)x^{3} } \right. \\ & \quad \quad - 4\left( { - 2 + \alpha } \right)\left( {5 + \alpha \left( {3 + 8\alpha } \right)} \right)x^{4} \\ & \quad \quad + \frac{1}{5}\left( { - 825 + \alpha \left( {1298 + \alpha \left( { - 729 + 128\alpha } \right)} \right)} \right)x^{5} \\ & \quad \quad + \frac{1}{3}\left( {121 + \alpha \left( { - 242 + \alpha \left( { - 351 + 280\alpha } \right)} \right)} \right)x^{6} - \frac{3}{27}\left( { - 1 + \alpha } \right)\alpha^{2} \left( {15 + 2\alpha } \right)x^{7} \\ & \quad \quad + 2\alpha^{2} \left( {51 + \alpha \left( { - 31 + 8\alpha } \right)} \right)x^{8} + \frac{8}{9}\alpha^{2} \left( { - 1 + \alpha \left( {17 + 16\alpha } \right)} \right)x^{9} \\ & \quad \quad - \left. {\frac{8}{5}\alpha^{2} \left( {11 + \alpha \left( { - 11 + 12\alpha } \right)} \right)x^{10} - \frac{192}{11}\alpha^{4} x^{11} + \frac{16}{3}\alpha^{4} x^{12} + \frac{112}{13}\alpha^{4} x^{13} + \frac{16}{7}\alpha^{4} x^{14} } \right) \\ \end{aligned}$$

In the same way, the iterations $p^{2} :\left( {\underline{V}_{2} \left( {x,\alpha } \right), \bar{V}_{2} \left( {x,\alpha } \right)} \right)$ and $p^{3} :\left( {\underline{V}_{3} \left( {x,\alpha } \right), \bar{V}_{3} \left( {x,\alpha } \right)} \right)$ were also computed for calculating fuzzy approximate solutions which could not be stated here as the iterations contain very long expressions.

We approximate $\underline{F} \left( {x,\alpha } \right)$ and $\bar{F}\left( {x,\alpha } \right)$ by setting *p* = 1 and the obtained fuzzy solutions are given by

$$\begin{aligned} \underline{F} \left( {x,\alpha } \right) & = \underline{u} \left( {x,\alpha } \right) = \mathop \sum \limits_{i = 0}^{3} \underline{V}_{i} (x,\alpha ) \\ & = \left( {\left( {2 - \alpha } \right)^{2} \left( { \frac{1}{2}x^{6} + x^{5} - x^{3} + \frac{11}{32}x^{2} } \right) - \frac{11}{32}x^{2} + \alpha x + \alpha } \right) \\ & \quad + x^{2} \left( {\alpha^{2} x + \frac{1}{48}\alpha \left( {33 + \alpha \left( { - 92 + 11\alpha } \right)x^{3} - \frac{1}{64}\alpha \left( {161 + \alpha \left( { - 140 + 43\alpha } \right)} \right)x^{4} + \cdots } \right.} \right. \\ \end{aligned}$$

and

$$\begin{aligned} \bar{F}\left( {x,\alpha } \right) & = \bar{u}\left( {x, \alpha } \right) = \mathop \sum \limits_{i = 0}^{3} \bar{V}_{i} \left( {x,\alpha } \right) \\ & = \left( {\alpha^{2} \left( {\frac{1}{2}x^{6} + x^{5} - x^{3} + \frac{11}{32}x^{2} } \right) + \left( {2 - \alpha } \right)\left( {\frac{ - 11}{32}\left( {2 - \alpha } \right)x^{2} + x + 1} \right)} \right) \\ & \quad - \frac{1}{64}x^{2} \left( { - 64(2 + \alpha )^{2} x + \frac{16}{3}\left( {2 + \alpha } \right)\left( { - 35 + 23\alpha } \right)x^{3} + \cdots } \right. \\ \end{aligned}$$

### Error analysis

Absolute errors are computed as

$$\underline{E} \left( {x,\alpha } \right) = \left| {\alpha \left( {x + 1} \right) - \underline{u} \left( {x,\alpha } \right)} \right|$$

$$\bar{E}\left( {x,\alpha } \right) = \left| {\left( {2 - \alpha } \right)\left( {x + 1} \right) - \bar{u}\left( {x,\alpha } \right)} \right|$$

**Fig. 2:**
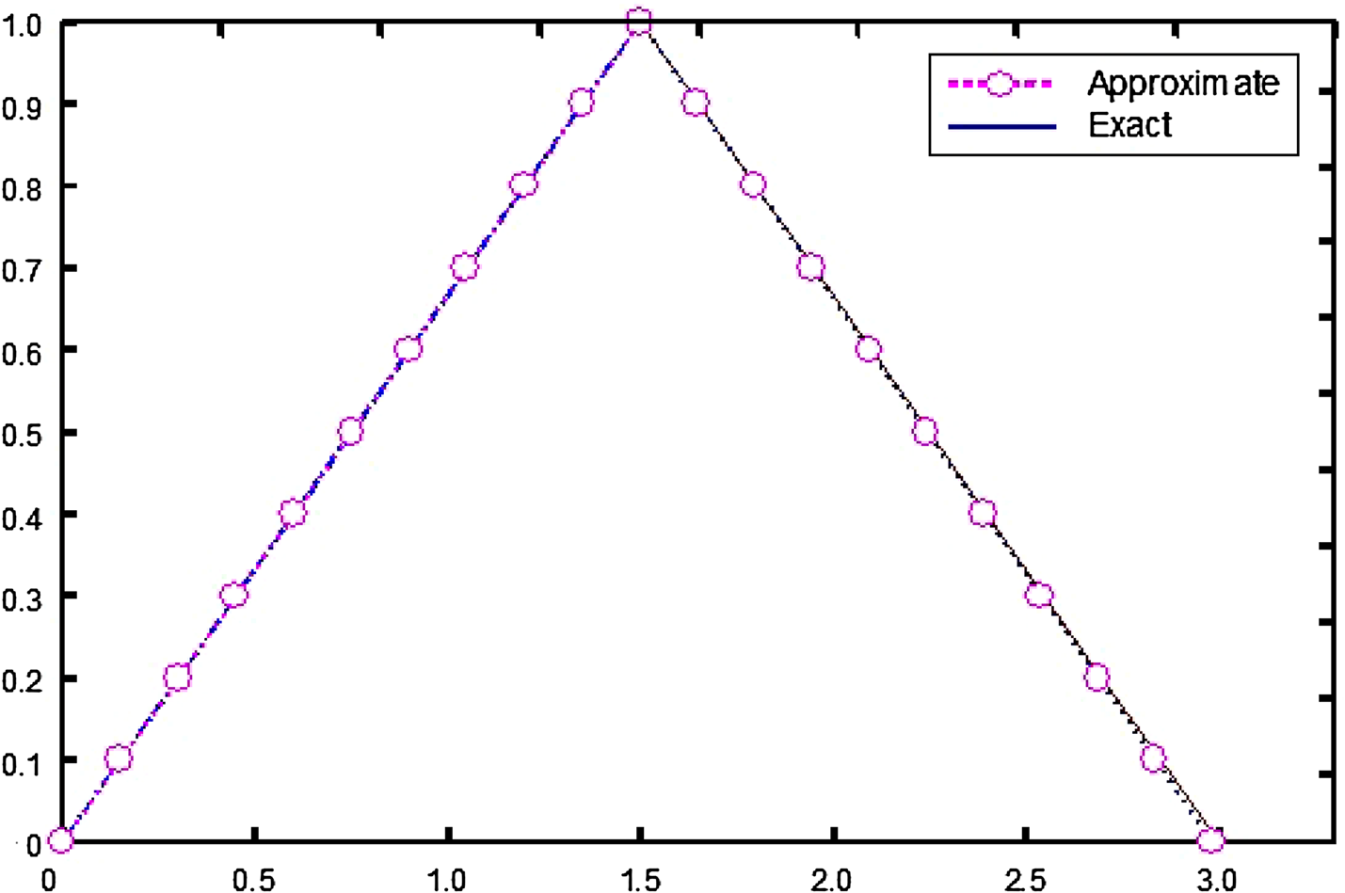
Plots of exact and approximate solutions of Example 2

By the same way of the previous example, we obtained the components of iteration formulae [Eq. (31)] by using the Mathematica program (Mathematica package version 5.2) according to the numerical algorithm where the tolerance is given a suitable positive value. The numerical results of the obtained approximate solutions are compared with the exact solutions for different *α***-**values and are presented in Table 2. Moreover exact and approximate solutions are shown graphically in Fig. 2.

**Table 2:** Exact and approximate solutions with four iterations at *x* = 0.5

*α*	Exact solution		Approximate solution		Error	
	$\underline{u} \left( {x,\alpha } \right)$	$\bar{u}\left( {x,\alpha } \right)$	$\underline{u} \left( {x,\alpha } \right)$	$\bar{u}\left( {x,\alpha } \right)$	$\underline{E} \left( {x,\alpha } \right)$	$\bar{E}\left( {x,\alpha } \right)$
0	0	3.00000	0	2.98221	0	0.01779
0.1	0.150000	2.85000	0.148744	2.83473	0.001256	0.01527
0.2	0.300000	2.70000	0.297259	2.68701	0.002741	0.01299
0.3	0.450000	2.55000	0.445361	2.53908	0.004639	0.01092
0.4	0.600000	2.40000	0.599574	2.39095	0.000426	0.00905
0.5	0.750000	2.25000	0.749317	2.24261	0.000683	0.00739
0.6	0.900000	2.10000	0.899174	2.09409	0.000826	0.00591
0.7	1.050000	1.95000	1.049622	1.94538	0.000378	0.00462
0.8	1.200000	1.80000	1.199686	1.79650	0.000314	0.00350
0.9	1.350000	1.65000	1.349564	1.64746	0.000436	0.00254
1	1.500000	1.50000	1.499300	1.49827	0.000700	0.00173

## Concluding remarks on numerical results

By looking into the computed values presented above (Tables 1, 2), one may conclude that the obtained approximate solutions by applying the proposed method (HPM) are in high agreement with the exact solutions. Furthermore, from the graphical representation (Figs. 1, 2), it is apparent that the plot of exact and approximate solutions merely coincide due to the occurrence of very minimum amount of errors ($\underline{E} \left( {x,\alpha } \right)$ and $\bar{E}\left( {x,\alpha } \right)$). The fuzzy approximate solutions are calculated at four iterations for the numerical examples considered. The results presented clearly shows that the iterative steps number 3–4 in the numerical algorithm accompany for the convergence of the fuzzy solution so that the computations are minimized and the accuracy of the approximate values is also achieved with exceptionally few iteration and overall errors are reduced.

## Conclusions

In this paper, we developed a simple yet versatile numerical technique using HPM and successfully applied it by means of the proposed algorithm for solving the linear and nonlinear fuzzy Volterra integral equations of the second kind. Mathematica has been used for computations in this paper. The examples analyzed illustrates that the answers are trusty and reveals the effectiveness of the proposed method. The reliability of HPM due to its precise results and the reduction in computation gives HPM a wider applicability. Moreover, it avoids the tedious work needed by the traditional numerical methods. Advantage of the proposed method lies in the free selection of initial approximation in a straightforward manner and also it overcomes the drawbacks of handling larger equations. This method does not involve any discretization of variables and hence it is free from rounding off errors. One can apply this method to higher order equations also.

Further research can be focused on studying the existence and convergence of HPM for linear and nonlinear FVIE-2. Finally we conclude that this work provides the applicable computational technique will aid the practical applications already used in the design of various fuzzy dynamical systems and it is an issue of considerable importance at this current trend due to its versatile nature.

## Abbreviations

HPM: homotopy perturbation method
FVIE-2: fuzzy Volterra integral equation of the second kind
